# Ortho­rhom­bic modification of (*E*)-4-benzyl­idene-2-phenyl-1,3-oxazol-5(4*H*)-one: whole mol­ecule disorder

**DOI:** 10.1107/S1600536809025999

**Published:** 2009-07-15

**Authors:** Seik Weng Ng

**Affiliations:** a Department of Chemistry, University of Malaya, 50603 Kuala Lumpur, Malaysia

## Abstract

The title mol­ecule, C_16_H_11_NO_2_, is disordered about a pseudo-twofold rotation axis that approximately bis­ects the mol­ecule along the C=O double bond. The two overlapping components are planar [r.m.s. deviation = 0.10 Å in the major 0.537 (4) component and 0.07 Å in the minor component]. The two components are aligned at 1.8 (3)°.

## Related literature

For the monoclinic modification, see: Busetti *et al.* (1993 ▶).
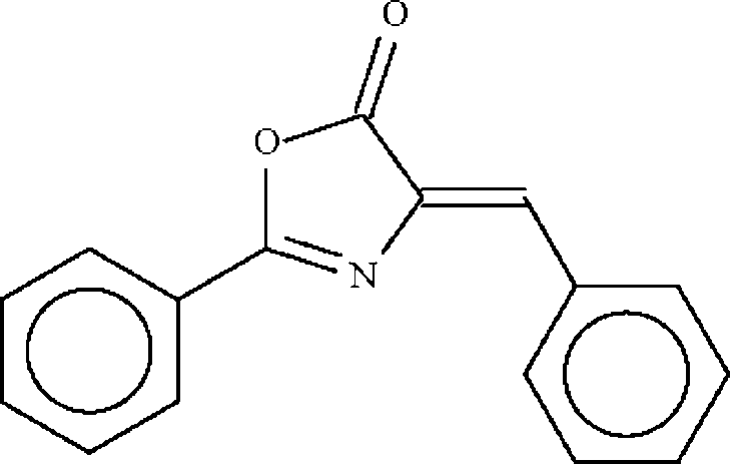


## Experimental

### 

#### Crystal data

C_16_H_11_NO_2_
*M* *_r_* = 249.26
Orthorhombic, 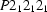

*a* = 3.9320 (1) Å
*b* = 14.7692 (5) Å
*c* = 20.6690 (6) Å
*V* = 1200.30 (6) Å^3^
*Z* = 4
Mo *K*α radiation
μ = 0.09 mm^−1^
*T* = 140 K
0.45 × 0.10 × 0.05 mm

#### Data collection

Bruker SMART APEX diffractometer
Absorption correction: none
8204 measured reflections
1640 independent reflections
1312 reflections with *I* > 2σ(*I*)
*R*_int_ = 0.036

#### Refinement

*R*[*F* ^2^ > 2σ(*F* ^2^)] = 0.044
*wR*(*F* ^2^) = 0.125
*S* = 1.03
1640 reflections
182 parameters
H-atom parameters constrained
Δρ_max_ = 0.29 e Å^−3^
Δρ_min_ = −0.17 e Å^−3^

### 

Data collection: *APEX2* (Bruker, 2008 ▶); cell refinement: *SAINT* (Bruker, 2008 ▶); data reduction: *SAINT*; program(s) used to solve structure: *SHELXS97* (Sheldrick, 2008 ▶); program(s) used to refine structure: *SHELXL97* (Sheldrick, 2008 ▶); molecular graphics: *X-SEED* (Barbour, 2001 ▶); software used to prepare material for publication: *publCIF* (Westrip, 2009 ▶).

## Supplementary Material

Crystal structure: contains datablocks global, I. DOI: 10.1107/S1600536809025999/tk2490sup1.cif

Structure factors: contains datablocks I. DOI: 10.1107/S1600536809025999/tk2490Isup2.hkl

Additional supplementary materials:  crystallographic information; 3D view; checkCIF report

## Figures and Tables

**Fig. 1.**
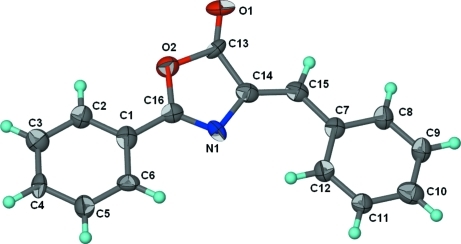
Thermal ellipsoid plot (Barbour, 2001) of the major, 0.537 (4), component of disordered C16H11NO2, shown at the 70% probability level. Hydrogen atoms are drawn as spheres of arbitrary radius.

**Fig. 2.**
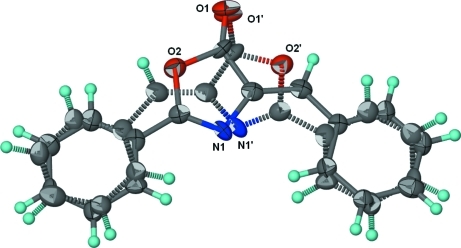
Detail showing the disorder. The minor disorder component is indicated with primed atoms and dotted bonds.

## supplementary crystallographic information

### Experimental

Anhydrous sodium acetate (0.26 g, 0.0036 mol) was added to solution of benzaldehyde (1 g, 0.0036 mol) and hippuric acid (0.77 g, 0.0043 mol) in acetic anhydride (0.27 ml, 0028 mol). The mixture was heated to 353 K for 2 h. Ethanol (10 ml) was added to the cool mixture to precipitate a yellow solid. This was collected and recrystallized from aqueous acetone to give yellow crystals in 60% yield; m.p. 443 K.

### Refinement

The molecule is disordered about a false 2-fold rotation axis that approximately bisects the molecule along the carbon-oxygen double bond. The aromatic rings were refined as rigid hexagons of 1.39 Å sides. The displacement factors of the primed atoms were restrained to those of the umprimed ones.

Carbon-bound H-atoms were placed in calculated positions (C—H 0.95 Å) and were included in the refinement in the riding model approximation with *U*(H) fixed at 1.2*U*_eq_(C).

In the absence of significant anomalous scattering effects, 1076 Friedel pairs were averaged in the final refinement.

### Crystal data

**Table:**

C_16_H_11_NO_2_	*F*(000) = 520
*M**_r_* = 249.26	*D*_x_ = 1.379 Mg m^−^^3^
Orthorhombic, *P*2_1_2_1_2_1_	Mo *K*α radiation, λ = 0.71073 Å
Hall symbol: P 2ac 2ab	Cell parameters from 1737 reflections
*a* = 3.9320 (1) Å	θ = 2.5–25.8°
*b* = 14.7692 (5) Å	µ = 0.09 mm^−^^1^
*c* = 20.6690 (6) Å	*T* = 140 K
*V* = 1200.30 (6) Å^3^	Prism, yellow
*Z* = 4	0.45 × 0.10 × 0.05 mm

### Data collection

**Table:**

Bruker SMART APEX diffractometer	1312 reflections with *I* > 2σ(*I*)
Radiation source: fine-focus sealed tube	*R*_int_ = 0.036
graphite	θ_max_ = 27.5°, θ_min_ = 1.7°
ω scans	*h* = −5→4
8204 measured reflections	*k* = −19→19
1640 independent reflections	*l* = −25→26

### Refinement

**Table:**

Refinement on *F*^2^	Secondary atom site location: difference Fourier map
Least-squares matrix: full	Hydrogen site location: inferred from neighbouring sites
*R*[*F*^2^ > 2σ(*F*^2^)] = 0.044	H-atom parameters constrained
*wR*(*F*^2^) = 0.125	*w* = 1/[σ^2^(*F*_o_^2^) + (0.0752*P*)^2^ + 0.1226*P*] where *P* = (*F*_o_^2^ + 2*F*_c_^2^)/3
*S* = 1.03	(Δ/σ)_max_ = 0.001
1640 reflections	Δρ_max_ = 0.29 e Å^−^^3^
182 parameters	Δρ_min_ = −0.17 e Å^−^^3^
0 restraints	Absolute structure: nd
Primary atom site location: structure-invariant direct methods	

### Fractional atomic coordinates and isotropic or equivalent isotropic displacement parameters (Å^2^)

**Table:**

	*x*	*y*	*z*	*U*_iso_*/*U*_eq_	Occ. (<1)
O1	−0.109 (5)	0.373 (3)	0.2237 (16)	0.031 (2)	0.537 (4)
O2	0.1605 (8)	0.2739 (2)	0.28782 (13)	0.0282 (5)	0.537 (4)
N1	0.432 (3)	0.3613 (3)	0.3629 (6)	0.0229 (10)	0.537 (4)
C1	0.4568 (11)	0.19591 (18)	0.37320 (16)	0.0256 (7)	0.537 (4)
C2	0.3888 (11)	0.1143 (2)	0.34231 (13)	0.0280 (10)	0.537 (4)
H2	0.2715	0.1139	0.3021	0.034*	0.537 (4)
C3	0.4924 (12)	0.03323 (18)	0.37021 (15)	0.0304 (9)	0.537 (4)
H3	0.4460	−0.0225	0.3491	0.037*	0.537 (4)
C4	0.6641 (13)	0.03378 (19)	0.42900 (16)	0.0236 (10)	0.537 (4)
H4	0.7349	−0.0216	0.4481	0.028*	0.537 (4)
C5	0.7321 (13)	0.1154 (2)	0.45989 (15)	0.0285 (11)	0.537 (4)
H5	0.8494	0.1158	0.5001	0.034*	0.537 (4)
C6	0.6284 (12)	0.19646 (18)	0.43199 (17)	0.0246 (9)	0.537 (4)
H6	0.6749	0.2522	0.4531	0.029*	0.537 (4)
C7	0.4345 (10)	0.5780 (2)	0.35423 (15)	0.0225 (7)	0.537 (4)
C8	0.4110 (11)	0.6682 (2)	0.33534 (13)	0.0264 (9)	0.537 (4)
H8	0.3029	0.6835	0.2957	0.032*	0.537 (4)
C9	0.5458 (14)	0.73583 (17)	0.37442 (17)	0.0291 (9)	0.537 (4)
H9	0.5297	0.7974	0.3615	0.035*	0.537 (4)
C10	0.7040 (15)	0.7134 (2)	0.43239 (17)	0.0333 (13)	0.537 (4)
H10	0.7960	0.7597	0.4591	0.040*	0.537 (4)
C11	0.7274 (14)	0.6233 (3)	0.45127 (17)	0.0255 (10)	0.537 (4)
H11	0.8356	0.6080	0.4909	0.031*	0.537 (4)
C12	0.5927 (12)	0.55561 (18)	0.41219 (18)	0.0270 (10)	0.537 (4)
H12	0.6088	0.4940	0.4251	0.032*	0.537 (4)
C13	0.081 (5)	0.3626 (7)	0.2675 (10)	0.0237 (18)	0.537 (4)
C14	0.2749 (12)	0.4188 (3)	0.31640 (18)	0.0247 (5)	0.537 (4)
C15	0.2789 (12)	0.5102 (3)	0.31202 (19)	0.0255 (6)	0.537 (4)
H15	0.1616	0.5341	0.2757	0.031*	0.537 (4)
C16	0.3550 (11)	0.2805 (3)	0.34289 (17)	0.0234 (6)	0.537 (4)
O1'	−0.037 (6)	0.376 (3)	0.2199 (18)	0.031 (2)	0.463
O2'	0.1691 (10)	0.4755 (2)	0.28423 (15)	0.0282 (5)	0.463
N1'	0.421 (4)	0.3896 (4)	0.3594 (7)	0.0229 (10)	0.463
C1'	0.4779 (13)	0.5543 (2)	0.36785 (19)	0.0256 (7)	0.463
C2'	0.4019 (13)	0.6351 (3)	0.33667 (16)	0.0280 (10)	0.463
H2'	0.2789	0.6345	0.2971	0.034*	0.463 (4)
C3'	0.5060 (16)	0.7170 (2)	0.3634 (2)	0.0304 (9)	0.463
H3'	0.4540	0.7723	0.3420	0.037*	0.463 (4)
C4'	0.6860 (18)	0.7179 (2)	0.4212 (2)	0.0236 (10)	0.463
H4'	0.7571	0.7738	0.4395	0.028*	0.463 (4)
C5'	0.7620 (17)	0.6370 (3)	0.4524 (2)	0.0285 (11)	0.463
H5'	0.8850	0.6377	0.4920	0.034*	0.463 (4)
C6'	0.6580 (15)	0.5552 (2)	0.4257 (2)	0.0246 (9)	0.463
H6'	0.7099	0.4999	0.4470	0.029*	0.463 (4)
C7'	0.4147 (13)	0.1731 (2)	0.35940 (18)	0.0225 (7)	0.463
C8'	0.3876 (14)	0.0824 (3)	0.34221 (15)	0.0264 (9)	0.463
H8'	0.2839	0.0660	0.3024	0.032*	0.463 (4)
C9'	0.5123 (17)	0.0157 (2)	0.38330 (19)	0.0291 (9)	0.463
H9'	0.4938	−0.0463	0.3716	0.035*	0.463 (4)
C10'	0.6641 (17)	0.0397 (3)	0.44157 (19)	0.0333 (13)	0.463
H10B	0.7493	−0.0059	0.4696	0.040*	0.463 (4)
C11'	0.6911 (16)	0.1304 (3)	0.45875 (19)	0.0255 (10)	0.463
H11B	0.7948	0.1468	0.4986	0.031*	0.463 (4)
C12'	0.5664 (15)	0.1971 (2)	0.4177 (2)	0.0270 (10)	0.463
H12B	0.5849	0.2591	0.4294	0.032*	0.463 (4)
C13'	0.112 (6)	0.3856 (9)	0.2697 (12)	0.0237 (18)	0.463
C14'	0.2695 (14)	0.3313 (3)	0.3171 (2)	0.0247 (5)	0.463
C15'	0.2625 (14)	0.2393 (3)	0.3151 (2)	0.0255 (6)	0.463
H15B	0.1408	0.2141	0.2797	0.031*	0.463 (4)
C16'	0.3654 (13)	0.4697 (3)	0.3398 (2)	0.0234 (6)	0.463

### Atomic displacement parameters (Å^2^)

**Table:**

	*U*^11^	*U*^22^	*U*^33^	*U*^12^	*U*^13^	*U*^23^
O1	0.017 (7)	0.048 (3)	0.029 (3)	−0.004 (7)	−0.006 (5)	0.000 (2)
O2	0.0293 (11)	0.0299 (10)	0.0255 (10)	−0.0017 (12)	−0.0039 (9)	−0.0011 (10)
N1	0.0248 (13)	0.021 (3)	0.0229 (14)	0.007 (4)	0.0002 (11)	0.006 (3)
C1	0.0230 (17)	0.0248 (14)	0.0290 (17)	0.0008 (17)	0.0078 (14)	−0.0006 (12)
C2	0.0316 (18)	0.027 (3)	0.0250 (15)	−0.001 (3)	0.0010 (12)	−0.0002 (14)
C3	0.032 (2)	0.0311 (17)	0.0284 (16)	0.001 (2)	0.0008 (15)	0.0019 (14)
C4	0.027 (3)	0.0140 (17)	0.0296 (17)	−0.004 (2)	0.0084 (16)	0.0016 (13)
C5	0.030 (2)	0.0227 (18)	0.033 (3)	0.0016 (19)	0.0003 (19)	−0.0007 (14)
C6	0.0154 (19)	0.0302 (19)	0.0281 (17)	0.0010 (17)	−0.0032 (15)	−0.0020 (14)
C7	0.0216 (16)	0.0235 (15)	0.0224 (13)	0.0007 (17)	0.0047 (12)	−0.0011 (12)
C8	0.0321 (19)	0.022 (2)	0.0253 (16)	0.000 (2)	0.0020 (14)	0.0007 (14)
C9	0.035 (2)	0.0214 (13)	0.0310 (17)	−0.0025 (18)	0.0019 (16)	0.0021 (12)
C10	0.030 (3)	0.042 (3)	0.0281 (18)	0.007 (3)	−0.0007 (18)	−0.0018 (17)
C11	0.0223 (19)	0.0247 (17)	0.030 (2)	0.000 (2)	−0.0010 (17)	0.0008 (15)
C12	0.027 (2)	0.0239 (18)	0.030 (2)	0.0014 (18)	−0.0006 (17)	−0.0007 (14)
C13	0.022 (4)	0.024 (6)	0.0252 (15)	−0.009 (5)	−0.002 (2)	−0.007 (5)
C14	0.0217 (13)	0.0305 (13)	0.0217 (12)	0.0035 (16)	0.0004 (11)	−0.0006 (14)
C15	0.0237 (15)	0.0306 (14)	0.0221 (13)	0.0036 (16)	0.0002 (12)	0.0011 (15)
C16	0.0222 (14)	0.0279 (14)	0.0202 (12)	−0.0010 (17)	0.0009 (11)	−0.0006 (15)
O1'	0.017 (7)	0.048 (3)	0.029 (3)	−0.004 (7)	−0.006 (5)	0.000 (2)
O2'	0.0293 (11)	0.0299 (10)	0.0255 (10)	−0.0017 (12)	−0.0039 (9)	−0.0011 (10)
N1'	0.0248 (13)	0.021 (3)	0.0229 (14)	0.007 (4)	0.0002 (11)	0.006 (3)
C1'	0.0230 (17)	0.0248 (14)	0.0290 (17)	0.0008 (17)	0.0078 (14)	−0.0006 (12)
C2'	0.0316 (18)	0.027 (3)	0.0250 (15)	−0.001 (3)	0.0010 (12)	−0.0002 (14)
C3'	0.032 (2)	0.0311 (17)	0.0284 (16)	0.001 (2)	0.0008 (15)	0.0019 (14)
C4'	0.027 (3)	0.0140 (17)	0.0296 (17)	−0.004 (2)	0.0084 (16)	0.0016 (13)
C5'	0.030 (2)	0.0227 (18)	0.033 (3)	0.0016 (19)	0.0003 (19)	−0.0007 (14)
C6'	0.0154 (19)	0.0302 (19)	0.0281 (17)	0.0010 (17)	−0.0032 (15)	−0.0020 (14)
C7'	0.0216 (16)	0.0235 (15)	0.0224 (13)	0.0007 (17)	0.0047 (12)	−0.0011 (12)
C8'	0.0321 (19)	0.022 (2)	0.0253 (16)	0.000 (2)	0.0020 (14)	0.0007 (14)
C9'	0.035 (2)	0.0214 (13)	0.0310 (17)	−0.0025 (18)	0.0019 (16)	0.0021 (12)
C10'	0.030 (3)	0.042 (3)	0.0281 (18)	0.007 (3)	−0.0007 (18)	−0.0018 (17)
C11'	0.0223 (19)	0.0247 (17)	0.030 (2)	0.000 (2)	−0.0010 (17)	0.0008 (15)
C12'	0.027 (2)	0.0239 (18)	0.030 (2)	0.0014 (18)	−0.0006 (17)	−0.0007 (14)
C13'	0.022 (4)	0.024 (6)	0.0252 (15)	−0.009 (5)	−0.002 (2)	−0.007 (5)
C14'	0.0217 (13)	0.0305 (13)	0.0217 (12)	0.0035 (16)	0.0004 (11)	−0.0006 (14)
C15'	0.0237 (15)	0.0306 (14)	0.0221 (13)	0.0036 (16)	0.0002 (12)	0.0011 (15)
C16'	0.0222 (14)	0.0279 (14)	0.0202 (12)	−0.0010 (17)	0.0009 (11)	−0.0006 (15)

### Geometric parameters (Å, °)

**Table:**

O1—C13	1.18 (4)	O1'—C13'	1.19 (5)
O2—C16	1.375 (5)	O2'—C13'	1.378 (13)
O2—C13	1.412 (15)	O2'—C16'	1.386 (6)
N1—C16	1.300 (9)	N1'—C16'	1.270 (8)
N1—C14	1.424 (9)	N1'—C14'	1.365 (14)
C1—C2	1.3900	C1'—C2'	1.3900
C1—C6	1.3900	C1'—C6'	1.3900
C1—C16	1.453 (4)	C1'—C16'	1.446 (6)
C2—C3	1.3900	C2'—C3'	1.3900
C2—H2	0.9500	C2'—H2'	0.9500
C3—C4	1.3900	C3'—C4'	1.3900
C3—H3	0.9500	C3'—H3'	0.9500
C4—C5	1.3900	C4'—C5'	1.3900
C4—H4	0.9500	C4'—H4'	0.9500
C5—C6	1.3900	C5'—C6'	1.3900
C5—H5	0.9500	C5'—H5'	0.9500
C6—H6	0.9500	C6'—H6'	0.9500
C7—C8	1.3900	C7'—C8'	1.3900
C7—C12	1.3900	C7'—C12'	1.3900
C7—C15	1.463 (5)	C7'—C15'	1.467 (6)
C8—C9	1.3900	C8'—C9'	1.3900
C8—H8	0.9500	C8'—H8'	0.9500
C9—C10	1.3900	C9'—C10'	1.3900
C9—H9	0.9500	C9'—H9'	0.9500
C10—C11	1.3900	C10'—C11'	1.3900
C10—H10	0.9500	C10'—H10B	0.9500
C11—C12	1.3900	C11'—C12'	1.3900
C11—H11	0.9500	C11'—H11B	0.9500
C12—H12	0.9500	C12'—H12B	0.9500
C13—C14	1.515 (15)	C13'—C14'	1.41 (2)
C14—C15	1.352 (5)	C14'—C15'	1.359 (6)
C15—H15	0.9500	C15'—H15B	0.9500
			
C16—O2—C13	107.7 (8)	C13'—O2'—C16'	102.2 (11)
C16—N1—C14	103.4 (8)	C16'—N1'—C14'	107.9 (10)
C2—C1—C6	120.0	C2'—C1'—C6'	120.0
C2—C1—C16	119.6 (3)	C2'—C1'—C16'	119.4 (3)
C6—C1—C16	120.4 (3)	C6'—C1'—C16'	120.6 (3)
C3—C2—C1	120.0	C3'—C2'—C1'	120.0
C3—C2—H2	120.0	C3'—C2'—H2'	120.0
C1—C2—H2	120.0	C1'—C2'—H2'	120.0
C2—C3—C4	120.0	C2'—C3'—C4'	120.0
C2—C3—H3	120.0	C2'—C3'—H3'	120.0
C4—C3—H3	120.0	C4'—C3'—H3'	120.0
C5—C4—C3	120.0	C5'—C4'—C3'	120.0
C5—C4—H4	120.0	C5'—C4'—H4'	120.0
C3—C4—H4	120.0	C3'—C4'—H4'	120.0
C6—C5—C4	120.0	C4'—C5'—C6'	120.0
C6—C5—H5	120.0	C4'—C5'—H5'	120.0
C4—C5—H5	120.0	C6'—C5'—H5'	120.0
C5—C6—C1	120.0	C5'—C6'—C1'	120.0
C5—C6—H6	120.0	C5'—C6'—H6'	120.0
C1—C6—H6	120.0	C1'—C6'—H6'	120.0
C8—C7—C12	120.0	C8'—C7'—C12'	120.0
C8—C7—C15	117.4 (3)	C8'—C7'—C15'	116.8 (4)
C12—C7—C15	122.5 (3)	C12'—C7'—C15'	123.1 (4)
C9—C8—C7	120.0	C7'—C8'—C9'	120.0
C9—C8—H8	120.0	C7'—C8'—H8'	120.0
C7—C8—H8	120.0	C9'—C8'—H8'	120.0
C8—C9—C10	120.0	C8'—C9'—C10'	120.0
C8—C9—H9	120.0	C8'—C9'—H9'	120.0
C10—C9—H9	120.0	C10'—C9'—H9'	120.0
C11—C10—C9	120.0	C11'—C10'—C9'	120.0
C11—C10—H10	120.0	C11'—C10'—H10B	120.0
C9—C10—H10	120.0	C9'—C10'—H10B	120.0
C10—C11—C12	120.0	C10'—C11'—C12'	120.0
C10—C11—H11	120.0	C10'—C11'—H11B	120.0
C12—C11—H11	120.0	C12'—C11'—H11B	120.0
C11—C12—C7	120.0	C11'—C12'—C7'	120.0
C11—C12—H12	120.0	C11'—C12'—H12B	120.0
C7—C12—H12	120.0	C7'—C12'—H12B	120.0
O1—C13—O2	119 (2)	O1'—C13'—O2'	113 (3)
O1—C13—C14	140 (2)	O1'—C13'—C14'	138 (3)
O2—C13—C14	101.4 (13)	O2'—C13'—C14'	109.0 (15)
C15—C14—N1	129.4 (5)	C15'—C14'—N1'	131.3 (6)
C15—C14—C13	120.5 (7)	C15'—C14'—C13'	122.6 (7)
N1—C14—C13	110.1 (8)	N1'—C14'—C13'	106.1 (7)
C14—C15—C7	130.4 (4)	C14'—C15'—C7'	129.7 (5)
C14—C15—H15	114.8	C14'—C15'—H15B	115.2
C7—C15—H15	114.8	C7'—C15'—H15B	115.2
N1—C16—O2	117.3 (6)	N1'—C16'—O2'	114.7 (8)
N1—C16—C1	126.0 (6)	N1'—C16'—C1'	128.5 (8)
O2—C16—C1	116.7 (4)	O2'—C16'—C1'	116.7 (4)
			
C6—C1—C2—C3	0.0	C6'—C1'—C2'—C3'	0.0
C16—C1—C2—C3	178.5 (4)	C16'—C1'—C2'—C3'	179.5 (5)
C1—C2—C3—C4	0.0	C1'—C2'—C3'—C4'	0.0
C2—C3—C4—C5	0.0	C2'—C3'—C4'—C5'	0.0
C3—C4—C5—C6	0.0	C3'—C4'—C5'—C6'	0.0
C4—C5—C6—C1	0.0	C4'—C5'—C6'—C1'	0.0
C2—C1—C6—C5	0.0	C2'—C1'—C6'—C5'	0.0
C16—C1—C6—C5	−178.5 (4)	C16'—C1'—C6'—C5'	−179.5 (5)
C12—C7—C8—C9	0.0	C12'—C7'—C8'—C9'	0.0
C15—C7—C8—C9	178.2 (4)	C15'—C7'—C8'—C9'	−177.1 (5)
C7—C8—C9—C10	0.0	C7'—C8'—C9'—C10'	0.0
C8—C9—C10—C11	0.0	C8'—C9'—C10'—C11'	0.0
C9—C10—C11—C12	0.0	C9'—C10'—C11'—C12'	0.0
C10—C11—C12—C7	0.0	C10'—C11'—C12'—C7'	0.0
C8—C7—C12—C11	0.0	C8'—C7'—C12'—C11'	0.0
C15—C7—C12—C11	−178.1 (4)	C15'—C7'—C12'—C11'	176.9 (5)
C16—O2—C13—O1	−173.9 (18)	C16'—O2'—C13'—O1'	−175 (2)
C16—O2—C13—C14	3.3 (13)	C16'—O2'—C13'—C14'	1.1 (18)
C16—N1—C14—C15	−178.0 (5)	C16'—N1'—C14'—C15'	177.7 (7)
C16—N1—C14—C13	2.4 (13)	C16'—N1'—C14'—C13'	−0.4 (16)
O1—C13—C14—C15	−7(3)	O1'—C13'—C14'—C15'	−4(4)
O2—C13—C14—C15	176.8 (7)	O2'—C13'—C14'—C15'	−178.8 (9)
O1—C13—C14—N1	173 (3)	O1'—C13'—C14'—N1'	175 (3)
O2—C13—C14—N1	−3.6 (15)	O2'—C13'—C14'—N1'	−1(2)
N1—C14—C15—C7	−2.2 (10)	N1'—C14'—C15'—C7'	1.2 (13)
C13—C14—C15—C7	177.3 (10)	C13'—C14'—C15'—C7'	179.0 (13)
C8—C7—C15—C14	176.7 (4)	C8'—C7'—C15'—C14'	−175.1 (5)
C12—C7—C15—C14	−5.2 (6)	C12'—C7'—C15'—C14'	7.9 (8)
C14—N1—C16—O2	−0.2 (11)	C14'—N1'—C16'—O2'	1.2 (14)
C14—N1—C16—C1	179.5 (5)	C14'—N1'—C16'—C1'	−179.1 (6)
C13—O2—C16—N1	−2.2 (12)	C13'—O2'—C16'—N1'	−1.4 (15)
C13—O2—C16—C1	178.1 (10)	C13'—O2'—C16'—C1'	178.8 (12)
C2—C1—C16—N1	−172.6 (8)	C2'—C1'—C16'—N1'	176.8 (10)
C6—C1—C16—N1	5.9 (9)	C6'—C1'—C16'—N1'	−3.7 (11)
C2—C1—C16—O2	7.1 (5)	C2'—C1'—C16'—O2'	−3.5 (6)
C6—C1—C16—O2	−174.4 (3)	C6'—C1'—C16'—O2'	176.0 (4)
